# Factors of microinflammation in non-diabetic chronic kidney disease: a pilot study

**DOI:** 10.1186/s12882-020-01803-y

**Published:** 2020-04-21

**Authors:** Valerie Olivier, Catherine Dunyach-Remy, Pierre Corbeau, Jean-Paul Cristol, Thibault Sutra, Stephane Burtey, Jean-Philippe Lavigne, Olivier Moranne

**Affiliations:** 1 Department of Nephrology – Dialysis – Apheresis, Caremeau Hospital, University Montpellier-Nîmes, CHU Nîmes, Nimes, France; 2 Department of Microbiology and Hospital Hygiene, U1047, INSERM, University of Montpellier, CHU Nîmes, Nîmes, France; 3 UMR9002, Institute for Human Genetics, CNRS-University of Montpellier, Montpellier, France; 4 PhyMedExp, INSERM, CNRS, University of Montpellier, Montpellier, France; 5 Department of Biochemistry and Hormonology, CHU Montpellier, Montpellier, France; 6 C2VN, INSERM 1263, INRA 1260, Aix-Marseille University, Marseille, France; 7 EA2415, Laboratoire Epidémiologie, Santé Publique, Biostatistiques, University of Montpellier, Nîmes, France

**Keywords:** CKD, Microinflammation, Oxidative stress, Uremic toxins, Bacterial translocation

## Abstract

**Background:**

The relationships between digestive bacterial translocation, uremic toxins, oxidative stress and microinflammation in a population of chronic kidney disease (CKD) patients without metabolic nor inflammatory disease are unknown.

**Methods:**

Bacterial translocation, uremic toxins, oxidative stress, and inflammation were assessed by measuring plasma levels of 16S  ribosomal DNA (16S rDNA), p-cresyl sulfate (PCS), indoxyl sulfate (IS), indole acetic acid (IAA), F2-isoprostanes, hsCRP and receptor I of TNFα (RITNFα) in patients without metabolic nor inflammatory disease. 44 patients with CKD from stage IIIB to V and 14 controls with normal kidney function were included from the nephrology outpatients. 11 patients under hemodialysis (HD) were also included. Correlations between each factor and microinflammation markers were studied.

**Results:**

16S rDNA levels were not increased in CKD patients compared to controls but were decreased in HD compared to non-HD stage V patients (4.7 (3.9–5.3) vs 8.6 (5.9–9.7) copies/μl, *p* = 0.002). IS, PCS and IAA levels increased in HD compared to controls (106.3 (73.3–130.4) vs 3.17 (2.4–5.1) μmol/l, *p* < 0.0001 for IS; 174.2 (125–227.5) vs 23.7 (13.9–52.6) μmol/l, *p* = 0.006 for PCS; and 3.7 (2.6–4.6) vs 1.3 (1.0–1.9) μmol/l, *p* = 0.0002 for IAA). Urea increased in non-HD stage V patients compared to controls (27.6 (22.7–30.9) vs 5.4 (4.8–6.4) mmol/l, *p* < 0.0001) and was similar in HD and in non-HD stage V (19.3 (14.0–24.0) vs 27.6 (22.7–30.9) mmol/l, *p* = 0.7). RITNFα levels increased in HD patients compared to controls (12.6 (9.6–13.3) vs 1.1 (1.0–1.4) ng/ml, p < 0.0001); hsCRP levels increased in non-HD stage V patients compared to controls (2.9 (1.4–8.5) vs 0.8 (0.5–1.7) mg/l, *p* = 0.01) and remained stable in HD patients (2.9 (1.4–8.5) vs 5.1 (0.9–11.5) mg/l, *p* = 1). F2-isoprostanes did not differ in CKD patients compared to controls. Among uremic toxins, IS and urea were correlated to RITNFα (r = 0.8, *p* < 0.0001 for both). PCS, IS and urea were higher in patients with hsCRP≧5 mg/l (*p* = 0.01, 0.04 and 0.001 respectively). 16S rDNA, F2-isoprostanes were not correlated to microinflammation markers in our study.

**Conclusions:**

In CKD patients without any associated metabolic nor inflammatory disease, only PCS, IS, and urea were correlated with microinflammation. Bacterial translocation was decreased in patients under HD and was not correlated to microinflammation.

## Background

Decreased glomerular filtration rate has been described as an independent factor associated with a high risk of cardiovascular events and mortality even at mild to moderate stages of chronic kidney disease (CKD) [1]. In addition to the high prevalence of traditional cardiovascular risk factors as diabetes mellitus and hypertension in CKD patients, microinflammation could contribute to accelerated atherosclerosis, as in general population [2].

Microinflammation in CKD results from many determinants apart from decreased clearance of proinflammatory cytokines: frequent infections [3], chronic heart failure [4], membrane bioincompatibility in hemodialysis (HD) [5], lack of vitamin D [6], metabolic acidosis [7], oxidative stress, uremic toxins, insulin resistance [8] and associated dysimmune comorbidities. More recently, the gut has been described as a contributor to microinflammation in CKD [9]. The kidney-gut concept illustrates the complex relationships between gut, uremic toxins and microinflammation in CKD: Gut microbiota undergoes modifications in CKD due to increased gut intraluminal urea, leading to selection of urease and uricase expressing bacteria and bacteria possessing indole and p-cresol-forming enzymes [10]. These bacteria will produce tryptophan or phenylalanine-derived uremic toxins such as indoxyl sulfate (IS), indole acetic acid (IAA) and p-cresol sulfate further sulfated into p-cresyl sulfate (PCS), which are known to have proinflammatory effects [11]. Increased intraluminal urea leads also to altered intestinal permeability and therefore to bacterial translocation that will result in activation of immune system and microinflammation [9]. Oxidative stress in CKD is mainly explained by an increase of reactive oxygen species production by activation and upregulation of pathways like nicotinamide adenin dinucleotide phosphatase (NADPH) oxidase [12] and Nox (NADPH oxidase) 4, mitochondria respiratory chain, xanthine oxidase, lipoxygenases, uncoupled nitric oxide synthases, the dysfunction of anti-oxidant systems due to the loss of nephrons [13]. CKD is associated with increased levels of oxidative stress markers such as oxidized nucleic acid, protein and lipid at different stages [14, 15]. Metabolic conditions such as insulin resistance and obesity, that are frequently present in CKD patients can even more enhance oxidative stress [12] and bacterial translocation [16, 17] and thus participate in microinflammation.

The relationships between bacterial translocation, uremic toxins, oxidative stress and microinflammation in a population of CKD patients are still unknown and need to be better described. The objective of the study is to understand the relationships between bacterial translocation, oxidative stress, uremic toxins and microinflammation in a selected population of consecutive patients from stage IIIB to end-stage kidney disease, without any associated metabolic nor inflammatory disease. The hypothesis is that, in CKD and in HD, enhanced bacterial translocation, oxidative stress and increased uremic toxins stimulate microinflammation.

## Methods

### Population

From December 2016 to June 2017, we consecutively enrolled all adult patients in the Department of Nephrology-Dialysis of the Nîmes University Hospital (France) for CKD from stage IIIB to V. HD patients were recruited in the same period in our department. A control population with normal kidney function were included from the nephrology outpatients followed up for an inactive nephrolithiasis disease.

The exclusion criteria were the presence of factors that may influence the inflammatory status or the bacterial translocation such as: diabetes mellitus, a body mass index (BMI) more than 30 kg/m^2^, a liver failure, a left ventricular ejection fraction below 40%, a history of inflammatory bowel disease, bariatric surgery or cancer, a recent history of chronic inflammatory disease, a current infection or a recent antibiotic treatment, a current immunosuppressive or immunomodulatory treatment, and a vascular catheter.

### Sociodemographic and clinical data

Epidemiological and clinical data were gathered for all patients including age, sex, body mass index, blood pressure, history of cardiovascular events, inflammatory disease, treatment, causes of CKD, active consumption of tobacco and the Charlson comorbidity index. For HD patients the following clinical data were collected: dialysis vintage, ultrafiltration volume, intradialytic hypotension defined as a fall of 40 mmHg of systolic blood pressure (SBP) or a SBP under 100 mmHg or the presence of symptoms suggesting hypotension during the current HD session, residual renal function, dialysis procedure (hemodialysis or hemodiafiltration).

### Biological data

The following biological data were gathered: creatinine and estimated glomerular filtration rate using the CKD EPI formula, urea, urinary albumin creatinine ratio, blood hemoglobin, natural vitamin D, and albuminemia.

### Blood samples

Remnant plasma and serum were obtained after routine exams from blood samples collected at the regular follow up visit for non-HD patients, and at the regular monthly blood analysis for HD patients. For HD patients, pre-HD blood samples were collected at the mid-week dialysis session. Blood was collected after a fasting period of at least 4 h, and patients were asked not to brush their teeth before blood collection to avoid physiological bacterial translocation. Plasma and serum were collected in EDTA-anticoagulant-treated tubes and dry tubes respectively after centrifugation and then stored at − 80 °C.

### DNA extraction and 16S rDNA real-time PCR

DNA was extracted from 200 μl of plasma using the *EZ1® DNA Tissue kit* (Qiagen, Courtaboeuf, France) according to the manufacturer’s recommendations. DNA was eluted in a 100 μl final volume. A negative control was extracted from molecular biology grade pure water in the same conditions. Bacterial 16S rDNA levels were measured by qPCR as described previously [18]. The assays were performed using a LightCycler 480 II (Roche). Absolute quantification analysis was performed with the Lightcycler 480 software (Roche), version 1.5, according to the manufacturer’s recommendations.

### HsCRP, RITNFα

The concentration of plasma hsCRP was determined by immuno-turbidimetry. Receptor I of TNFα (RITNFα) plasmatic rates were measured as an inflammation marker with *Quantikine® ELISA Human TNF RI/TNFRSF1A* ELISA kit (R&D SYTEMS, Minneapolis, United States). The dilution factor was 1:20 according to the manufacturer’s recommendations.

### Uremic toxins

Plasmatic PCS, IAA and IS were measured by high performance liquid chromatography as described previously [19].

### Oxidative stress

Plasmatic F2-Isoprostanes were measured by chromatography-mass spectrometry as marker of oxidative stress [20].

### Statistical analysis

Quantitative data are presented as median (interquartile range). Categorical data are given in frequency and percentage. Markers of bacterial translocation, oxidative stress, inflammation, uremic toxins (IS, PCS, IAA) and urea are expressed according to CKD stage as follows: CKD stage IIIB, CKD stage IV, CKD stage V and CKD stage V under HD. Due to the low number of patients in each group and the non-Gaussian distribution of the variables, non-parametric tests were used. Quantitative values according to CKD were compared as follows: bivariate group comparisons were performed with a Mann-Whitney test or a global test of Kruskal-Wallis with a multipair wise comparison from Dunn when more than 2 groups were compared. For categorical data a Chi squared test was used to compare groups. The correlations between bacterial translocation, urea, uremic toxins, oxidative stress and RITNFα as continuous variables were analyzed with a Spearman test. Considering hsCRP as a categorical data, we compared the bacterial translocation, urea, uremic toxins and oxidative stress levels in subjects with hsCRP ≥5 mg/l with those in subjects with hsCRP < 5 mg/l. Statistical difference was set up at a *p*-value < 0.05 for bilateral test. Statistical analysis was performed using GraphPad Prism version 7.

## Results

### Demographic and clinical characteristics

**Fig. 1:**
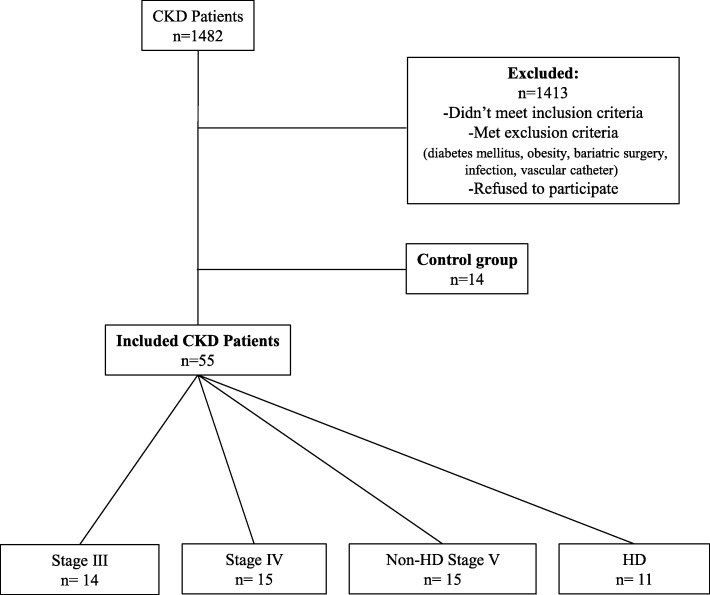
Selection of the study population

Sixty-nine patients were included in this study: 14 patients in the CKD stage IIIB group, 15 patients in the stage IV group and non-HD stage V group, 11 patients in the HD group and 14 patients in the control group (Fig. 1). The clinical characteristics and standard biological parameters are detailed in Table 1. No statistical difference in term of clinical characteristics could be observed among the different groups except for BMI (*p* = 0.02), SBP (*p* = 0.04), and Charlson comorbidity index (*p* < 0.0001). BMI and systolic blood pressure were low in HD group and Charlson comorbidity index was particularly high in stage V group, HD and non-HD.

**Table 1:** Baseline characteristics of population

	Controls						Stage IIIB						Stage IV						Stage V						Stage V-HD						
			n = 14						n = 14						n = 15						n = 15						n = 11				p
**Clinical data**																															
Age, years, median [IQ]	56.0	[	47.5	–	64.0	]	60.0	[	52.3	–	69.0	]	64.0	[	58.5	–	67.5	]	53.0	[	49.5	–	68.5	]	58.0	[	34.0	–	74.0	]	0.7
% Males	57.1						78.6						73.3						80.0						36.4						0.1
BMI^a^, kg/m^2^, median [IQ]	23.0	[	22.0	–	25.9	]	27.4	[	26.5	–	28.5	]	24.6	[	23.2	–	26.5	]	25.4	[	23.4	–	28.7	]	21.9	[	20.8	–	24.6	]	**0.02**
SBP^b^, mmHg, median [IQ]	125.0	[	117.8	–	131.5	]	129.0	[	123.3	–	143.0	]	143.0	[	137.0	–	152.5	]	140.0	[	129.0	–	156.0	]	118.0	[	107.0	–	142.5	]	**0.04**
DBP^c^, mmHg, median [IQ]	84.5	[	77.0	–	91.3	]	85.5	[	78.8	–	88.5	]	84.0	[	78.5	–	92.0	]	86.0	[	76.0	–	90.0	]	70.0	[	67.5	–	79.0	]	0.1
**Comorbidities, n (%)**																															
Smoker	2.0	(	14.3	%	)		2.0	(	14.3	%	)		6.0	(	40.0	%	)		4.0	(	26.7	%	)		5.0	(	45.5	%	)		0.3
Previous cardiovascular comorbidities	0.0	(	0.0	%	)		4.0	(	28.6	%	)		4.0	(	26.7	%	)		5.0	(	33.3	%	)		5.0	(	45.5	%	)		0.1
Charlson comorbidity index [IQ]	1.0	[	0.0	–	2.0	]	1.5	[	1.0	–	3.0	]	2.0	[	1.0	–	4.0	]	4.0	[	3.0	–	5.0	]	4.0	[	2.0	–	5.0	]	**< 0.0001**
**Etiologies, n(%)**																															
Glomerular disease			–				3.0	(	21.4	%	)		4.0	(	26.7	%	)		6.0	(	40.0	%	)		5.0	(	45.5	%	)		0.5
Renovascular			–				6.0	(	42.9	%	)		7.0	(	46.7	%	)		4.0	(	26.7	%	)		2.0	(	18.2	%	)		0.4
ADPKD^d^			–				3.0	(	21.4	%	)		3.0	(	20.0	%	)		4.0	(	26.7	%	)		0.0	(	0.0	%	)		0.3
Urological			–				1.0	(	7.1	%	)		0.0	(	0.0	%	)		0.0	(	0.0	%	)		2.0	(	18.2	%	)		0.2
Other			–				1.0	(	7.1	%	)		1.0	(	6.7	%	)		1.0	(	6.7	%	)		2.0	(	18.2	%	)		0.7
**Biological Data**																															
Creatinine, μmol/l, median [IQ]	78.0	[	68.8	–	84.8	]	168.5	[	148.8	–	176.5	]	251.0	[	225.5	–	303.5	]	437.0	[	365.5	–	518.0	]	657.0	[	645.0	–	890.5	]	**< 0.0001**
GFR, ml/min/1,73m^2^ (CKD EPI), median [IQ]	90.5	[	81.3	–	94.3	]	36.0	[	33.3	–	39.8	]	20.0	[	17.0	–	22.5	]	11.0	[	9.5	–	12.0	]	–						**< 0.0001**
ACR^e^, mg/mmol, median [IQ]	0.8	[	0.5	–	1.6	]	2.6	[	2.3		15.9	]	70.0	[	16.7	–	157.9	]	157.6	[	49.8	–	216.5	]	–						**< 0.0001**
Urea, mmol/l, median [IQ]	5.4	[	4.9	–	6.1	]	9.6	[	8.8	–	11.7	]	16.8	[	13.3	–	19.6	]	27.6	[	23.15	–	30.5	]	19.3	[	14.5	–	23.0	]	**< 0.0001**
Hemoglobin, g/dl, median [IQ]	14.4	[	13.4	–	15.6	]	13.8	[	12.6	–	15.0	]	12.6	[	11.5	–	13.6	]	11.3	[	10.7	–	12.4	]	11.5	[	11.1	–	12.5	]	**< 0.0001**
PTH, pg/ml, median [IQ]	48.0	[	44.0	–	55.0	]	63.0	[	45.3	–	86.3	]	198.0	[	94.5	–	288.5	]	217.0	[	98.0	–	535.0	]	218.0	[	138.0	–	387.0	]	**< 0.0001**
D Vitamine, nmol/l, median [IQ]	64.0	[	54.0	–	93.0	]	84.0	[	68.5	–	106.3	]	69.0	[	44.0	–	81.0	]	65.0	[	50.0	–	105.0	]	107.5	[	70.8	–	123.3	]	0.2
Albumin, g/l, median [IQ]	49.0	[	46.9	–	50.6	]	45.9	[	44.3	–	47.2	]	44.5	[	43.8	–	47.4	]	46.8	[	43.4	–	47.5	]	42.8	[	42.3	–	45.1	]	**0.001**
**Dialytic parameters**																															
KT/V, median [IQ]			–						–						–						–				2.1	[	1.8	–	2.3	]	–
Residual clearance, ml/min, median [IQ]			–						–						–						–				0.0	[	0.0	–	1.1	]	–
Dialysis vintage, months, median [IQ]			–						–						–						–				51.0	[	43.5	–	122.0	]	–
Perdialytic hypotension, n(%)			–						–						–						–				1.0	(	9.1	%	)		–
Ultrafiltration, ml, median [IQ]			–						–						–						–				1700.0	[	1050.0	–	2500.0	]	–
Weekly duration. h. median [IQ]			–						–						–						–				12.0	[	12.0	–	12.0	]	–
Epuration mode, n(%)																															–
HD			–						–						–						–				2.0	(	18.2	%	)		–
HDF			–						–						–						–				9.0	(	81.8	%	)		–

^a^*BMI* body mass index, ^b^*SPB* systolic blood pressure, *DBP*^c^ diastolic blood pressure, *ADPKD*^d^: Adult dominant polycystic kidney disease, *ACR*^e^ urinary albumin / creatinine ratio

### Bacterial translocation, uremic toxins and oxidative stress according to CKD stage

**Fig 2:**
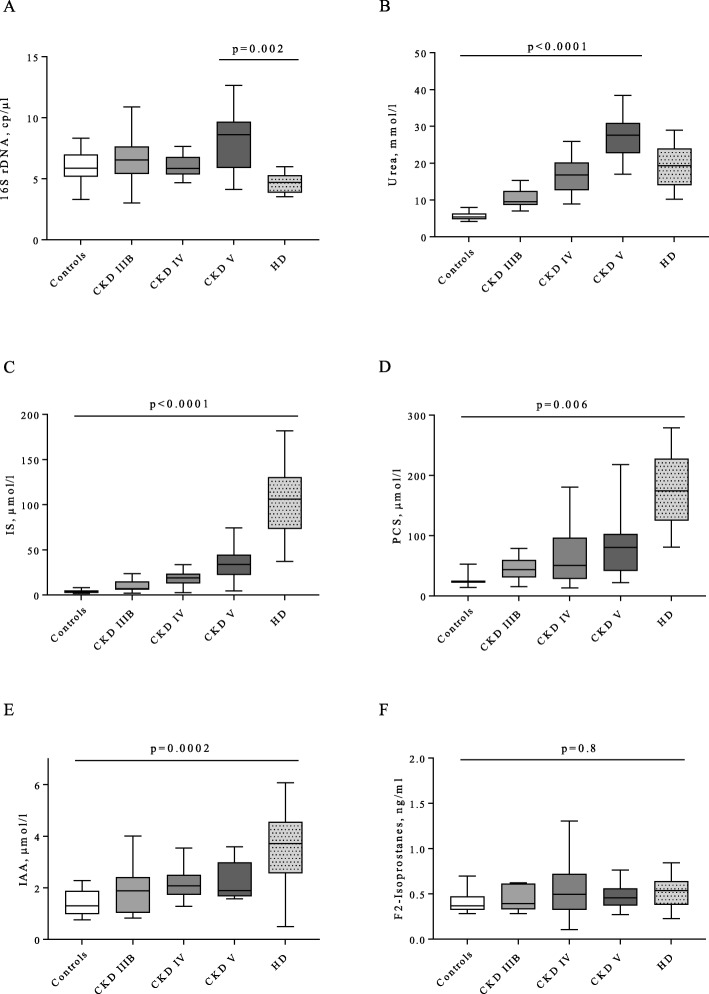
Bacterial translocation, urea, uremic toxins and oxidative stress according to CKD stage. **a**- Plasma 16S rDNA, copies/μl, **b**- plasma urea, mmol/l, **c**-plasma indoxyl sulfate (IS), μmol/l, **d**- plasma p-cresyl sulfate (PCS), μmol/l, **e**- plasma indole acetic acid (IAA), μmol/l, **f**- plasma F2-isoprostanes, ng/l. Kruskal-Wallis statistic test with multipaire wise comparison from Dunn: *n* = 14 for controls, n = 14 for stage IIIB, *n* = 15 for stage IV, n = 15 for non-HD stage V, *n* = 11 for HD except for PCS: *n* = 3 for controls, *n* = 12 for stage IIIB, n = 14 for stage IV, n = 14 for non-HD stage V, *n* = 9 for HD

For each CKD group, bacterial translocation was assessed by plasmatic 16S rDNA quantification, uremic toxins: IS, PCS, IAA and urea were measured, and oxidative stress was assessed by F2-isoprostanes plasma quantification (Fig. 2). 16S rDNA levels were not increased in non-HD CKD patients compared to controls (8.6 (5.9–9.7) in non-HD stage V vs 5.9 (5.2–7.0) copies/μl in controls, *p* = 0.4). Surprisingly, plasmatic 16S rDNA decreased in HD patients compared to non-HD stage V patients (4.7 (3.9–5.3) in HD patients vs 8.6 (5.9–9.7) copies/μl in non-HD stage V, *p* = 0.002) (Fig. 2a). Moreover, the levels of plasmatic 16S rDNA in HD patients were similar to those of controls.

Since urea has been described as a gut toxic in CKD, we analyzed its variations: urea displayed increasing levels in non-HD stage V patients compared to controls (27.6 (22.7–30.9) in non-HD stage V vs 5.4 (4.8–6.4) mmol/l in controls, *p* < 0.0001) and similar levels in HD compared to non-HD stage V (19.3 (14.0–24.0) in HD patients vs 27.6 (22.7–30.9) mmol/l in non-HD stage V, *p* = 0.7) (Fig. 2b). 16S rDNA levels were not correlated with urea (p = 0.7).

To evaluate the possible role of uremic toxins produced by the gut microbiota in bacterial translocation and in microinflammation in CKD, IS, PCS and IAA plasmatic levels were measured for each group. IS, PCS and IAA increased with the stage of CKD and in HD (Fig. 2c-e). IS, PCS and IAA levels were higher in HD than in controls (106.3 (73.3–130.4) in HD patients vs 3.2 (2.4–5.1) μmol/l in controls, *p* < 0.0001 for IS; 174.2 (125.0–227.5) in HD patients vs 23.7 (13.9–52.6) μmol/l in controls, *p* = 0.006 for PCS; and 3.7 (2.6–4.6) in HD patients vs 1.3 (1.0–1.9) μmol/l in controls, *p* = 0.0002 for IAA). To evaluate the role of oxidative stress in microinflammation, we measured plasmatic F2-isoprostanes. However, F2-isoprostanes levels did not vary with the stage of CKD nor in HD patients (Fig. 2f).

### Microinflammation according to CKD stage

**Fig. 3:**
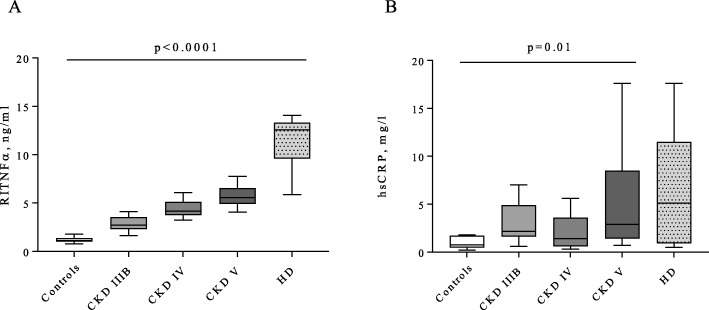
Microinflammation according to CKD stage. **a**- RITNFα, ng/l, **b**- hsCRP, mg/l. Kruskal-Wallis statistic test with multipaire wise comparison from Dunn: n = 14 for controls, n = 14 for stage IIIB, n = 15 for stage IV, n = 15 for non-HD stage V, n = 11 for HD

To evaluate if microinflammation was related to bacterial translocation, uremic toxins, or to oxidative stress, we measured hsCRP and RITNFα in each group. RITNFα levels were higher in HD patients than in controls (12.6 (9.6–13.3) in HD patients vs 1.1 (1.0–1.4) ng/ml in controls, *p* < 0.0001) and hsCRP levels were higher in non-HD stage V patients than in controls (2.9 (1.4–8.5) in non-HD stage V vs 0.8 (0.5–1.7) mg/l in controls, *p* = 0.01) and remained stable in HD patients (2.9 (1.4–8.5) in non-HD stage V vs 5.1 (0.9–11.5) mg/l in HD patients, *p* = 1) (Fig. 3a-b). Thus, microinflammation rather followed the course of uremic toxins IS, PCS and IAA, than that of bacterial translocation.

### Factors associated with microinflammation in CKD

**Fig 4:**
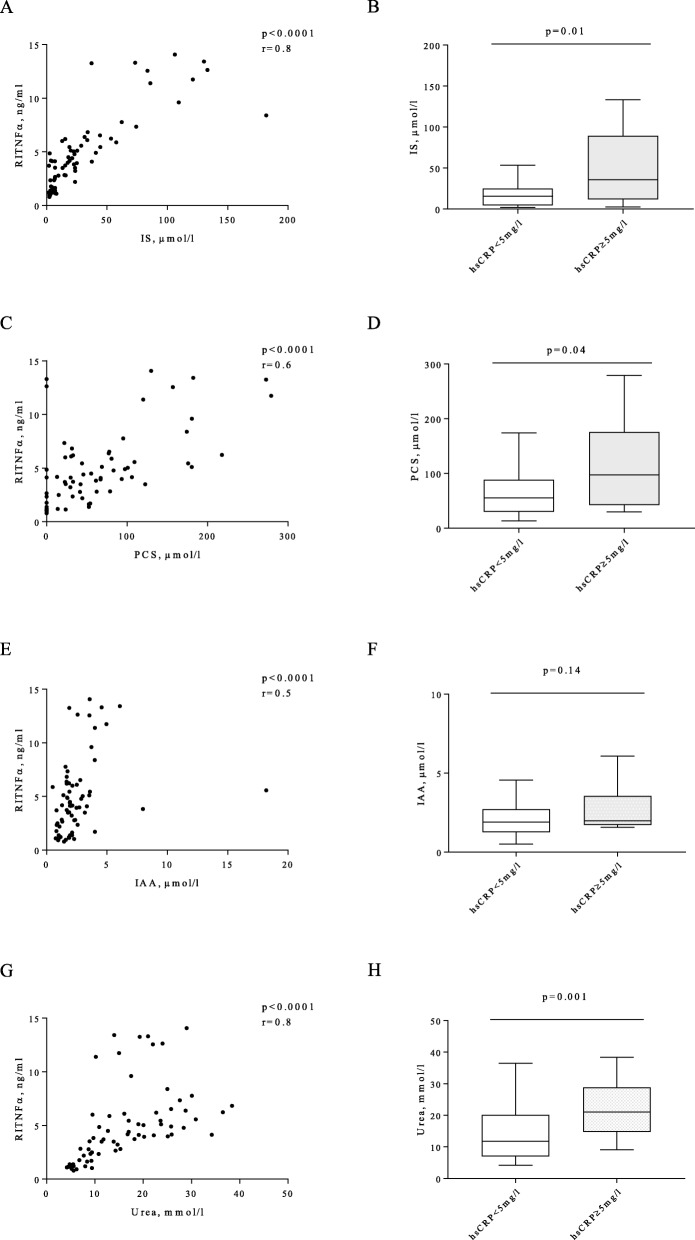
Factors associated with microinflammation in CKD. **a**- Correlation between indoxyl sulfate (IS) and RITNFα, **c**- correlation between p-cresyl sulfate (PCS) and RITNFα, **e**- correlation between indole acetic acid (IAA) and RITNFα, **g**- correlation between urea and RITNFα. **b**- IS levels in subgroups with hsCRP < or ≥ 5 mg/l. **d**- PCS levels in subgroups with hsCRP < or ≥ 5 mg/l. **f**- IAA levels in subgroups with hsCRP < or ≥ 5 mg/l. **h**- Urea levels in subgroups with hsCRP < or ≥ 5 mg/l. Spearman correlation test for A, C, E, G and Mann-Whitney test for B, D, F, H: *n* = 51 for hsCRP < 5 mg/l, *n* = 18 for hsCRP ≥5 mg/l, except for PCS: *n* = 37 for hsCRP < 5 mg/l, n = 15 for hsCRP ≥5 mg/l

Then we determined the associations between microinflammation and bacterial translocation, uremic toxins and oxidative stress. 16 s rDNA or F2-isoprostanes were not correlated to microinflammation in CKD. IS was correlated with RITNFα, (r = 0.8, *p* < 0.0001), PCS and IAA were also correlated with RITNFα but to a lesser extent (0.6 and 0.5 respectively, p < 0.0001) (Fig. 4a, c, e). IS and PCS were significantly higher in patients with hsCRP ≥5 mg/l than in patients with hsCRP< 5 mg/l (*p* = 0.01 and *p* = 0.04, respectively) (Fig. 4b, d). IAA levels were not significantly increased in patients with hsCRP ≥5 mg/l (Fig. 4f).

As IS, PCS and IAA are synthetized by proteolytic bacteria selected from the gut microbiota under high levels of intraluminal urea, we analyzed the association between plasmatic urea and microinflammation. Urea was correlated with RITNFα (r = 0.8, *p* < 0.0001) and was significantly higher in patients with hsCRP≥5 mg/l vs hsCRP< 5 mg/l (*p* = 0.01) (Fig. 4g, h), suggesting that urea could play a role in microinflammation in CKD.

## Discussion

This study highlights that CKD patients present increased microinflammation markers even in the absence of diabetes mellitus, obesity, active infections, neoplastic or auto immune diseases. This microinflammation increases with the stage of CKD and even more in HD patients as has been already shown in literature [21]. We found that only uremic toxins IS, PCS and urea were associated with microinflammation in CKD. Surprisingly, our CKD patients did not show any significant variation in the oxidative stress marker F2-isoprostanes compared to controls. Bacterial translocation marker was not increased in non-HD patients compared to controls, and clearly decreased in HD patients compared to non-HD stage V.

To our knowledge, our study is the first translational study showing a decrease of bacterial translocation in HD patients compared to non-HD advanced stages of CKD. HD patients could be at risk of increasing bacterial translocation due to per dialytic episodes of hypotension, ultrafiltration, mesenteric ischemia, impaired cardiac function, reduction of splanchnic blood flow that impair intestinal barrier [22]. Indeed, the study from McIntyre showed increased plasmatic rates of lipopolysaccharide in CKD patients and even more in patients under HD [9]. One reason that we found conflicting results is that we used plasmatic levels of bacterial 16S rDNA which allowed us to detect all translocated bacteria from intestine to blood specifically if measured after a fasting period. Another reason is that we excluded patients with catheter and hemodynamic instability as only one HD patient displayed a perdialytic hypotension during the analyzed session. According to these results, microinflammation in our HD patients is not related to bacterial translocation.

The level of bacterial translocation was not increased in our non-HD CKD patients compared to controls. The patients did not display any known confounding factors of bacterial translocation such as diabetes mellitus [16], obesity [17], cardiac failure, HIV infection [23], inflammatory bowel disease [24], alcohol disorder [18] which could favor gut barrier permeability and thus lead to high plasmatic 16S rDNA levels. Another point is that our CKD patients had a high vitamin D level, at any stage of the disease, probably due to an efficient supplementation. It has been shown that apart from its immune modulating effects, vitamin D influences digestive barrier quality. In vivo, it could restore gut permeability in a mouse model of colitis, acting on the tight junction proteins [25]. Furthermore, HIV and hepatitis C co-infected patients had a lower bacterial translocation if they had a 25-hydroxy vitamin D in the normal range compared to vitamin D deficient patients [26]. This could explain the fact that our patients had a low level of bacterial translocation. Another provocative hypothesis is that indoles could have a protective effect on the gut barrier. In a mouse model of colitis, low stool levels of IAA were associated with intestinal inflammation [27]. High plasmatic levels of tryptophan metabolites like IAA in stage V non-HD and HD patients could activate aryl hydrocarbon receptors which regulate local IL-22 production for intestinal homeostasis [28] resulting in gut barrier protection. Interestingly, a recent study evaluated blood microbiome in non-diabetic CKD patients using 16S PCR: quantitative circulating 16S rDNA did not differ between CKD patients and controls [29] supporting the fact that bacterial translocation is not increased in non-diabetic CKD patients. Of course, we also can not eliminate a lack of effect and a falsely unchanged rate of bacterial translocation, due to the low number of patients in this pilot study.

One key factor known to lead to the impairment of the gut barrier and to bacterial translocation in CKD is urea. Urea has been more described as a reflection of other toxic metabolites accumulation in CKD than a real uremic toxin. Albeit recently, urea has gained recognition as a toxic metabolite implied in endothelial and adipocyte dysfunction and in gut barrier impairment [30] as intraluminal urea increases in the intestine with decline of renal function. Urea is directly toxic on gut barrier [31], leading to endocytosis of tight junction proteins [31, 32] and to an increased gut permeability, and thus to an abnormal passage of bacteria or bacterial fragments from the gut lumen to the circulation [33], stimulating the immune system. In our study, plasmatic urea was not correlated to 16S rDNA suggesting non-linear and more complex relationship between plasmatic and intraluminal urea and bacterial translocation. Surprisingly, bacterial translocation was not correlated with microinflammation in our study whereas urea was, meaning that urea could participate in microinflammation in CKD by another mechanism than altering gut permeability and increasing bacterial translocation. Thus, one hypothesis is that urea could stimulate microinflammation by the selection of IS, PCS, IAA producing bacteria in gut microbiota but this hypothesis cannot be confirmed in this pilot study.

Our CKD patients did not show any significant increase in plasmatic F2-isoprostanes compared to controls. It is important to note that oxidative stress is difficult to assess in vivo. There is no reference marker to quantify oxidative stress in routine, and literature presents many different markers without any consensus [34]. Among all those markers, quantification of plasmatic F2-isoprostanes by mass spectrometry appeared to be the most reliable to measure oxidative stress associated with CKD in patients [35]. Interestingly, our study is the first, to our knowledge, to describe oxidative stress in a population of CKD patients, without metabolic diseases and without hemodynamic instability in HD. As a result, we cannot conclude that oxidative stress is a major determinant in microinflammation in CKD patients without metabolic comorbidities.

Our study showed a positive association between microinflammation markers and uremic toxins such as IS at any CKD stage. This toxin is known to take a part in immune and endothelial cell activation in CKD. IS was described as being a direct actor in atherosclerosis development, enhancing the production of microparticles by activated endothelial cells [36], and inhibiting in vitro the proliferation and reparation of endothelium [37, 38]. Furthermore IS could simulate the synthesis of proinflammatory cytokines by monocytes such as IL-6 and IL-1β [39]. Increased IS has been described as a risk factor of vascular calcification, arterial stiffness and cardiovascular mortality in a population of HD and non-HD CKD patients. In our work, the toxins IS, PCS and IAA were increased in CKD patients and even more in HD patients, suggesting that they are poorly eliminated in HD. These toxins are protein bound and can only be eliminated by adsorption in HD therefore other therapeutics aiming at decreasing these toxins can be meaningful. The spherical carbon adsorbent of indole AST-120 has been shown to reduce IS serum levels and improve uremic symptoms such as malaise [40] in CKD patients. Unfortunately AST-120 has shown no beneficial effect on the progression of CKD in the EPPIC trials [41].

The main limitations of our monocentric study are the cross sectional study design, the low number of subjects, limiting analyses with lack of power. The variability of samples did not allow us to obtain clear subgroup analysis especially for bacterial translocation, therefore we cannot eliminate a link between bacterial translocation and microinflammation in non-HD stage V. A longitudinal and more powerful study could be interesting to precise bacterial translocation evolution in CKD. However, our data allowed us to raise hypothesis about microinflammation caused only by reduced renal function in a strictly selected CKD population.

## Conclusions

We showed that CKD patients without any associated metabolic or inflammatory diseases presented high markers of inflammation, particularly in HD. Bacterial translocation was unchanged in CKD patients compared to controls but clearly decreased under HD treatment compared to late stage of CKD. Microbiota derived uremic toxins such as IS could definitely play an important role in microinflammation and atherosclerosis development in those patients.

## Data Availability

The datasets used during the current study are available from the corresponding author on reasonable request.

## Abbreviations

BMI: Body mass index
CKD: Chronic kidney disease
HD: Hemodialysis
IAA: Indole acetic acid
IS: Indoxyl sulfate
NADPH: Nicotinamide adenin dinucleotide phosphatase
PCS: P-cresyl sulfate
RITNFα: Receptor I of TNFα
SBP: Systolic blood pressure
